# Current Trends in Development and Use of Polymeric Ion-Exchange Resins in Wastewater Treatment

**DOI:** 10.3390/ma17235994

**Published:** 2024-12-06

**Authors:** Nicoleta Mirela Marin, Mihai Nita Lazar, Marcela Popa, Toma Galaon, Luoana Florentina Pascu

**Affiliations:** 1 National Research and Development Institute for Industrial Ecology-ECOIND, 57-73 Drumul Podu Dambovitei, 060652 Bucharest, Romania; nicoleta.marin@incdecoind.ro (N.M.M.); mihai.nita@incdecoind.ro (M.N.L.); marcela.popa@incdecoind.ro (M.P.); 2 Department of Oxide Materials Science and Engineering, National University of Science and Technology Politehnica Bucharest, 1–7 Gh. Polizu, 060042 Bucharest, Romania; 3 Department of Analytical and Physical Chemistry, University of Bucharest, 4-12 Regina Elisabeta Bd., 030018 Bucharest, Romania; 4 The Research Institute of the University of Bucharest, 90 Panduri Street, Sector 5, 050663 Bucharest, Romania

**Keywords:** ion-exchange resin, reutilization, pollutants, selectivity, water treatment

## Abstract

Drinking and wastewater are to be treated for safe human consumption and for keeping surface waters clean. There are multiple water purification procedures, but the use of ion-exchange resins significantly enhances water purification efficiency. This review was targeted on highlighting the concept and classification of polymeric ion-exchange resins as well as pointing out their real-world applications. Their successful use for purification purposes has been linked to their chemical structure, simplicity of operation, accessibility, and reusability. Therefore, polymeric ion-exchange resins have been used for the removal of a wide range of organic and inorganic pollutants such as pharmaceutical compounds, dyes, organic matter, metals, and many others. Ion-exchange resins are obtained directly by synthesis methods or grafting ionizable groups on polymer matrix in order to ensure continuous improvement. Furthermore, the newly designed ion-exchange resins take into consideration biodegradability potential towards obtaining ecofriendly compounds.

## 1. Introduction

Today, ion-exchange materials possess a large number of applications across different industries [1,2,3,4,5,6]. Their ability to selectively exchange ions makes them invaluable for water purification processes, where they can effectively remove unwanted metallic ions and organic contaminants [7,8,9,10]. Additionally, in fields like clean energy, these materials are used in batteries and fuel cells to enhance performance and efficiency [11,12,13]. In biomedical applications, such as bone tissue engineering, ion-exchange materials can facilitate the delivery of ions necessary for cellular functions [14,15,16]. The versatility and adaptability of ion-exchange technologies continue to drive innovations across many sectors, starting from highly polluted mining industry [10] to cleaning vital resources of drinking water. 

Ion exchangers are materials that facilitate the exchange of ions between a solution and the solid phase, playing a crucial role in various applications such as water treatment [7,8,17,18,19,20,21], metal recovery [7,21,22,23,24,25,26,27,28], pharmaceutical processes, etc. [29,30]. 

Ion exchangers are solid materials that can attract and hold ions within their structure, allowing for the replacement of these ions with others present in a solution. This process occurs through electrostatic interactions, where the exchange of ions leads to changes in the composition of the solution without significantly altering the physical form of the ion-exchange material [31,32,33,34,35,36,37]. The field of ion exchange is in continuous progress, generating novel materials and applications to solve complex environmental and industrial challenges [38,39,40,41,42,43]. 

Continuous research in this domain leads to more efficient and selective use of ion-exchange materials [44,45,46,47]. This review focuses on the fundamental concepts of ion exchangers, categorizing them based on their chemical structure, charge type, and real-world water-treatment applications. We focused also on biodegradation in order to emphasize the wastewater routine treatment processes from wastewater treatment plants (WWTPs). The biodegradation section clearly shows the role of microorganisms in environmental protection by biodegrading a wide range of chemical compounds. Actually, this biodegradation step is one of the most important unspecific treatment steps linked to WWTPs, which are essential in environmental protection. A huge diversity of chemical compounds ends up in domestic and industrial wastewaters, which are treated by WWTPs before releasing them into the environment. Among this chemical diversity, the ion-exchange compounds are present and, therefore, are biodegraded during the (bio)treatment steps. The biodegradation section pinpoints how this routine treatment procedure happens in domestic and industrial WWTPs.

## 2. Ion-Exchange Resins—General Aspects

The use of ion-exchange resins in wastewater treatment has many advantages because they could remove almost all cationic and anionic organic or inorganic pollutants. The popularity of ion-exchange resins has remained constant over the years because this separation method can be applied for environmental depollution (water, air, soil, and waste), biotechnology, agriculture, pharmaceuticals, and other industries. Another important advantage has been the wide variety of commercially available resins, some of which are specific for certain applications [38,39,48,49,50,51,52,53].

Ion exchange has been described as the oldest scientific phenomenon known to mankind. This claim originated from Bible descriptions [54] and writings from Aristotle, J. T. Way and H. S. Thompson where it was described that, in the soil, calcium was replaced by ammonium ions. This observation was the basis for further ion-exchange equilibrium studies as databases for ion-exchange resin development [36,37,55]. 

The first synthetic ion-exchange resins were obtained in 1935 by Adams and Holmes and were based on a phenol-formaldehyde structure, which was modified with sulfonic groups [37]. 

Subsequently, new research was focused on the use of ion-exchange resins obtained by the copolymerization of styrene with divinylbenzene. Herein are attached to the hydrocarbon structure, by synthetic methods, sulfonic or carboxyl groups to give cation exchangers and amine groups for the synthesis of anion exchangers. 

These types represent 90% of the resins used in analytical studies. Subsequently, cellulose-based ion exchangers, as well as ion exchangers containing complex-forming groups, were introduced [56].

Ion-exchange resins are macromolecular polyelectrolytes consisting of chemically inert, three-dimensional polymer networks (the resin matrix) on which are grafted acidic or basic functional groups that ensure the participation of the resin in the ion-exchange equilibrium. Chromatographic separation by an ion-exchange mechanism is based on electrostatic interactions between a counterion that neutralizes the functional groups of the resin and an ion of the same charges in the mixture to be separated [56].

## 3. Synthesis of Ion-Exchange Resins and Physico-Chemical Proprieties

In general, the synthesis of a modern ion exchanger is carried out in two steps. In the first step, the spherical pellet is obtained by the copolymerization of styrene with divinylbenzene in water and is currently the most widely used industrial process for the manufacture of polystyrene resins. This copolymerization step is also used in the synthesis of non-functionalized polymers used in sorption equilibrium.

In the second step of the synthesis, the ionic functional groups are grafted. These contain a fixed group that is non-exchangeable and a counterion that participates in the ion-exchange equilibrium. The functional groups may be acidic or basic and may be grafted onto the polymer substrate by synthetic methods or may pre-exist in the structure in one of the monomers [57,58]. 

**Figure 1:**
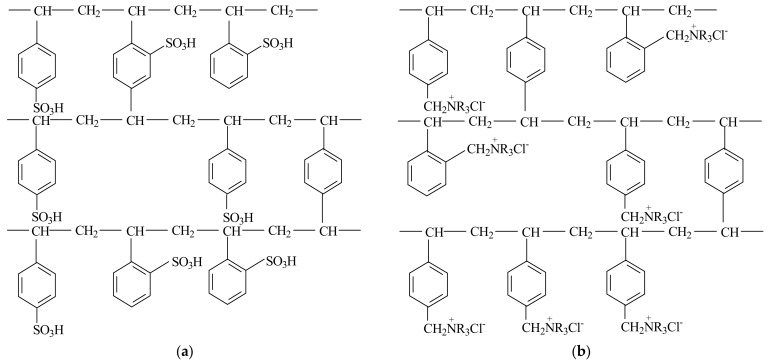
Structure of a cation-exchange resin with sulfonic groups (**a**) and anion-exchange resin with quaternary amine groups (**b**) (adapted according to reference [60]).

Cation-exchange resins have negatively charged ionizable functional groups and exchange positively charged counterions in the solution. The structures of cation and anion-exchange resins are shown in Figure 1a. 

Anion-exchange resins have positively charged ionizable functional groups and exchange negatively charged counterions in the solution (Figure 1b). 

Mixed-bed resins combine both cation and anion-exchange resins, allowing the efficient removal of both types of ions from the solution [59].

### 3.1. Specific Cases of Obtaining Ion Exchangers

**Figure 2:**
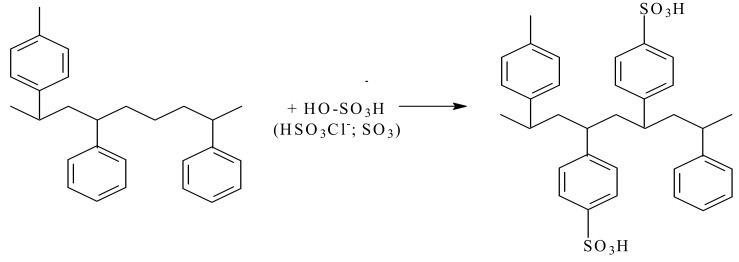
Synthesis of a strongly acidic cation exchanger based on styrene–divinylbenzene (adapted according to reference [62]).

A strongly acidic cation exchanger with -SO_3_H groups is obtained by grafting them onto the benzene ring in the styrene–divinylbenzene copolymer structure at 90–140 °C, being graphical represented in Figure 2 [61].

**Figure 3:**
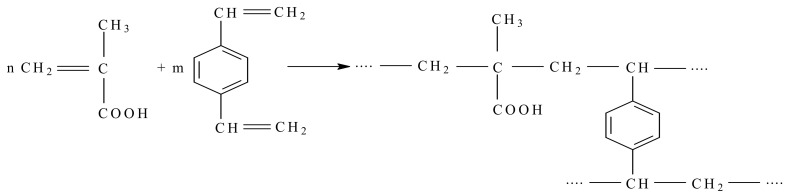
Synthesis of the polymer support for a weakly acidic cation-exchange resin (adapted according to reference [62]).

Some of the weakly acidic cations can be obtained from the copolymerization reaction of divinylbenzene with methacrylic acid, shown schematically in Figure 3.

**Figure 4:**
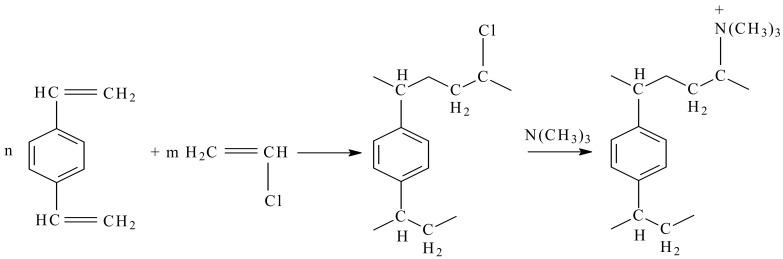
The reaction to obtain a strongly basic anion-exchange resin (adapted according to reference [62]).

Weakly and strongly basic anion-exchange resins are obtained by the copolymerization of divinylbenzene with vinyl chloride, followed by grafting of the amino groups according to the scheme in Figure 4.

### 3.2. Classification of Ion-Exchange Resins

**Figure 5:**
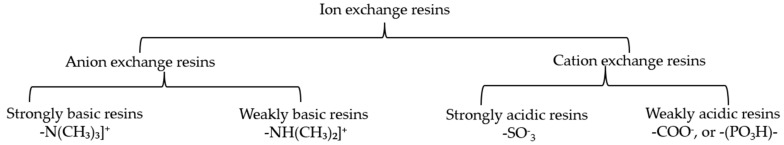
Classification of ion-exchange resins (adapted according to reference [63]).

Depending on the strength of the functional group grafted onto the hydrocarbon chain, ion-exchange resins are classified according to the scheme shown in Figure 5.

Cation-exchange resins can be classified according to the nature of the ionizable groups into strongly acidic or weakly acidic ion-exchange resins. Strongly acidic cations contain -SO^−^_3_H^+^ groups grafted onto the three-dimensional structure. Weakly acidic cations contain -COOH, or -(PO_3_H)-, groups resulting from the ionization of the -(PO_3_H)-H^+^ group, or -(PO_2_H)- groups resulting from the ionization of the -(PO_2_H)-H^+^ group, or -O^−^ H^+^ [64].

Anion-exchange resins can be classified according to the nature of the ionizable groups into strongly basic resins containing -N(CH_3_)_3_]^+^OH- grafted on the polymeric structure. Quaternary amine groups or weakly basic resins containing amine groups, tertiary -NH(CH_3_)_2_]^+^OH-, secondary -NH_2_(CH_3_)]^+^OH-, or primary -NH_3_]^+^ result from the ionization of -NH_3_]^+^OH- [65].

The chemical behavior of strong cation-exchange resins is similar to that of a strong acid. Therefore, the behavior of strong acid resins is not influenced by the pH of the solution. Weakly acidic cation-exchange resins behave similarly to their organic acids, which are weakly ionized.

For a weakly acidic resins, the ionizable group is typically a carboxylic acid, -COOH. The degree of the dissociation of a weakly acidic resins is strongly influenced by the pH of the solution. Strongly basic anion-exchange resins are ionized over the entire pH range and are used in Cl^−^ or HO^−^ form. Table 1 shows the main types of ion-exchange resins and the pH range in which they can be used [66]. 

### 3.3. Physical Properties of Ion-Exchange Resins

The increasing number of resins available allows the selection of a particular type of resin for each field of research. In this case, the most important physical properties of resins, swelling, particle size, the degree of cross-linking, have to be taken into account to select the optimal resin used in analytical separations.

### 3.4. Resin Behavior in the Presence of Water (Swelling)

Resin retains water according to the degree of cross-linking, functional groups, and ionic form. Water has low molecular mass and penetrates into the resin granule, swelling it and increasing its volume without dissolving it due to the polymeric structure, which is insoluble.

Ion-exchange resins containing acid or basic groups attached to their structure swell when immersed in water; this swelling is influenced by the percentage of divinylbenzene and the nature of the existing functional groups.

The swelling differs according to the ionic form, as follows: a cation-exchange resin that has the same degree of swelling and Na^+^ ions attached to each sulfonic group on the structure will have a significantly lower retention than the same resin in the H^+^ form [57,67]. Table 2 shows the percentages of water that can be retained as a function of the percentages of divinylbenzene (DVB) in the copolymer structure.

### 3.5. Resin Particle Size

The size of the resin particle influences the rate at which ion-exchange equilibrium is reached and is measured in standard units (mesh). A mesh represents the number of openings per square centimeter of sieve. Thus, smaller particles improve the speed at which ion-exchange equilibrium is reached in a shorter time necessary to reach equilibrium. On the other hand, the smaller particle offers a lower flow velocity through the column [57].

### 3.6. Degree of Cross-Linking

Divinyl benzene forms bonds with the styrene polymer chain contributing to the three-dimensional structure, making the resin insoluble in water and determining the extent to which it can swell or return to its original form by the removal of water.

The degree of cross-linking refers to the percentage of divinylbenzene that the resin contains; this is determined in the synthesis process. Thus, the lower the percentage of divinylbenzene, the more the copolymer will swell and the less resistant the resins. A low degree of cross-linking allows the exchange of high molecular weight ions. In contrast, copolymers with high divinylbenzene content swell poorly and have high stiffness and mechanical strength, as well as low porosity inside the resin. Resins with 4–10% divinylbenzene are used for most analytical applications [70].

### 3.7. Chemical Properties of Ion-Exchange Resins

#### Ion-Exchange Capacity (Cs)

The capacity of an ion-exchange resin is defined as the concentration of ionizable groups reported to 1 g of dry resin. Thus, the exchange capacity is attributed to sulphonic, carboxylic, phosphoric, phosphonic, and phenolic groups and in anion-exchange resins to amine, quaternary, tertiary, secondary, and primary groups. In both cases, ion exchange occurs not only at the surface but also within the pores of the resin structure. The ion-exchange capacity of resins is influenced by the rate of the diffusion of ions throughout the resin structure. Thus, an experimentally determined exchange capacity that is higher than that recommended by the resin manufacturer can be attributed to other interactions occurring in addition to the ion exchange. A low exchange capacity value may be determined when ions in the solution cannot reach the exchange centers of the resin due to steric factors preventing access to them. Exchange capacity is evaluated per unit volume of swollen resin, and experimental data are expressed in equivalent/L (eq/L) or milliequivalent/L (meq/L) [57,71].

The theoretical and operational exchange capacities of the ion-exchange resins are shown in Table 3 (the values shown were determined by titration) [68], where 1 equivalent (eq represents 1 mol per unit of charge exchanged, and 1 meq represents 0.001 moles per unit charge exchanged).

### 3.8. Resin Affinity

Resins have different affinities for ions in solution; thus, based on this behavior, activity series have been established for ion-exchange resins. In terms of studying the behavior of a strongly acidic cation-exchange resin, their affinity increases with the charge and the atomic number of ions found in the solution under analysis [57].

The affinity of resin is defined as its ability to have a preference for certain ions in the presence of other ions found in the solution. The cation-exchange resin has affinity for ions with high volume and charge. When solutions are concentrated, the resin has affinity for ions of small volume and charge. The affinity series for cation and anion-exchange resins, respectively, are described below [60,72,73].

The affinity sequence for a cation-exchange resin is described as follows [74]:

$$Hg^{2+}<Li^{+}<H^{+}<Na^{+}<K^{+}\approx NH_{4}^{+}<Cd^{2+}<Cs^{+}<Ag^{+}<Mn^{2+}<Mg^{2+}<Zn^{2+}<Cu^{2+}<Ni^{2+}<Co^{2+}<Ca^{2+}<Sr^{2+}<Pb^{2+}<Al^{3+}<Fe^{3+}$$

The affinity sequence for an anion-exchange resin is described as follows [75]:

HO^−^ ≈ F^−^ < HCO_3_^−^ < Cl^−^ < Br^−^ < NO_3_^−^ < HSO_4_^−^, PO_4_^3−^ < CrO_4_^2−^ < SO_4_^2−^

Based on the experimental data, for a strongly acidic cation-exchange resin of the Dowex 50 type and a solution containing monovalent cations of the same concentration, the affinity for ions is as follows [76]:

Ag^+^ > Tl^+^ > Cs^+^ > Rb^+^ > NH^+^_4_ > K^+^ > Na^+^ > Li^+^

### 3.9. Resin Regeneration

In most applications, when the charged ions on the resin exceed the exchange capacity of the resin, the resin is considered to be depleted and must be regenerated [17,77,78,79]. Thus, once the resin is depleted, it no longer retains ions in solution, which is monitored by a continuous measurement of effluent conductivity. Exhausted resin is regenerated with solutions in which the ion concentration must be high enough to remove the ions retained in the regenerated resin. In the case of ion-exchange resins, the regenerant may be a mineral acid or a concentrated salt solution [57,80]. An efficient regeneration of ion-exchange resins can reduce the cost and promote sustainability within industrial applications [81,82,83]. 

### 3.10. Ion-Exchange Equilibrium Study

For a strongly acidic cation-exchange resin, the ion-exchange equilibrium is described by the following reaction [49]:

R-Y^−^X^+^_(r)_ + C^+^_(s)_ <=> R-Y^−^C^+^_(r)_ + X^+^_(s)_

where R represents the hydrocarbon network; Y^-^ the functional group of the cation-exchange resin; X^+^ the exchangeable counterion of the functional group.

The ion-exchange equilibrium for a strongly basic anion-exchange resin is described by the following reaction:

R-X^+^B^−^_(r)_ + A^−^_(s)_ <=> R-X^+^A^−^_(r)_ + B^−^_(s)_

where R represents the hydrocarbon network; X^+^ the functional group of the anion-exchange resin; B^−^ the exchangeable counterion of the functional group.

### 3.11. Ion-Exchange Equilibrium Modeling of Pollutants on Polymeric Exchange Resins

When a cation-exchange resin is placed in contact with a solution containing different cations, the resin has different affinities for each cation, a behavior known as resin selectivity. 

Selectivity can be quantified experimentally based on ion-exchange isotherms, which describe the number of ions retained on the polymeric material under optimal conditions.

The ion-exchange isotherm is defined as the distribution of the ion found in the polymer ion exchanger *Q_e_* (mg/g) and its concentration in the solution obtained at equilibrium *C_e_* (mg/L). In those aims, graphical isotherm is constructed by plotting the ionic fraction at equilibrium vs. the amount of ion retained in the resin mass. So, a linear isotherm describes the case where the resin does not have affinity for any of the ions present in the solution. An ideal isotherm is obtained when the number of adsorbents increases with the initial concentration and, finally, when saturation is reached and the isotherm obtains a good selectivity for one or more ions present in the solution [84,85,86].

## 4. Application of Ion-Exchange Resin for Wastewater Depollution

### 4.1. Application of Ion-Exchange Resin for Azo Dye Removal

Azo dyes are an important category of chemical compounds that are widely used in many productions; they have become a major source of surface water pollution. Azo dyes contain in their structure azo groups (-N=N-) and an aromatic ring structure, which is in an anionic form helping their solubilization in water. Technologies used in the treatment of colored wastewater include physical, chemical, and biological methods [87]. Thus, adsorption methods widely used in the treatment of polluted wastewater have a high operational efficiency and the possibility of utilization in a new desorption adsorption cycle. Usually, low-cost materials have certain limitations in the operational processes and some difficulties in reuse. Consequently, ion-exchange resins are an important direction in wastewater treatment that can solve the different kinds of problems described above [88]. 

Azo dyes are toxic, mutagenic, and carcinogenic for the aquatic environment; thus, they must be removed from wastewater. The literature reports propose their removal using weakly basic anion-exchange resins like Amberlyst A21 (A21), which was used in the removal of three azo dyes, namely, C.I. Direct Red 23 (DR23), C.I. Direct Orange 26 (DO26), and C.I. Direct Black 22 (DB22), obtaining the following adsorption capacity determined by applying the Langmuir isotherm equation: 491.5 mg/g DB 22, 271.1 mg/g DR 23, and 285.6 mg/g DO 26. Resin regeneration was obtained using 1 M HCl and NaOH and also a mixture of 1 M NaCl in 50% *v/v* methanol (MeOH) [89].

In order to treat wastewater polluted with methylene blue (MB), Wang et al. obtained a new resin by the sulfonation of styrene resin with concentrated H_2_SO_4_ to obtain a new resin, namely, sodium polystyrene sulfonate (PSSNa). Subsequently, the sulfonic acid resin was involved in MB retention and showed a capacity at equilibrium of 16.9 mg/g adsorptive adsorption. PSSNa was subjected to five adsorptive/desorption cycles where its adsorptive capacity decreased by only 20% compared to the initial adsorption [90].

For the depollution of real textile wastewater (RTWW), Lewatit^®^ MonoPlus S 108 resin (strongly acidic) (SAC) and Lewatit^®^ MonoPlus M 500 strongly basic (quaternary ammonium type 1) (SAB) were employed in the technological process (Leverkusen, Germany). For the harmless ions Ca^2+^ and Mg^2+^ removal, SAC resin after coagulation and the flocculation process were applied. Also, for Cl^−^, SO_4_^2−^, and acid black 194 (anionic dye), SAB resin was used. The study showed that textile effluents polluted with high concentrations of salts and anionic dyes can be pretreated with high efficiency by ion exchange [91]. 

For the treatment of wastewater polluted with the dye Acid Orange 7, three anion-exchange resins were used, namely, weakly basic (Lewatit MonoPlus MP 62), intermediate (Lewatit MonoPlus MP 64), and strongly basic (Lewatit MonoPlus MP 500). The best adsorption was determined for the strongly basic resin. The adsorption capacity was also influenced by the temperature of the aqueous medium. 

For the desorption study, the following conditions were used: 1 M KSCN in MeOH (80–90%) for MP 62, and AO7 desorption was 95% to 100%; for MP 64 applying the same conditions, AO7 was desorbed up to 88%. Also, 1 M HCl in 90% MeOH was used for MP 500, and 87% AO7 was detected in the supernatant solution after the desorption study [92].

The adsorption of cationic dye from malachite green (MG) by the synthetic Diaion CR-11 chelating resin and Amberlite IRC-748 cation-exchange resin has been evaluated. The equilibrium results showed adsorption capacities of 102.1 mg/g for Diaion CR-11 and 480.6 mg/g for Amberlite IRC-748. It was concluded that the electrostatic interaction together with Van der Waals forces, π–π interaction, and hydrogen bonding can influence the adsorption of cationic dye [6].

Marin et al. used Amberlite IRA400, a strongly basic anion-exchange resin based on the styrene/divinylbenzene matrix for Gryfalan Navy Blue RL (GNB) removal. After adsorption, it was determined that the maximum adsorption capacity was 435 mg/g, taking into consideration the Langmuir model. Also, using the Dubinin–Radushkevich isotherm, the free energy of adsorption process was determined, and it was greater than 16 kJ/mol. The previous value suggests that the ion exchange was the main mechanism that governed the GNB adsorption together with physical adsorption given by the interactions π–π produced by the GNB and resin structure [33].

In another study, IRA 402(Cl^−^), a strongly basic anion exchanger in chloride form, was employed for Acid Blue 113 (AB 113) removal. For this study, experimental parameters that affect the ion-exchange process were evaluated. Also, it was found that the adsorption capacity of anion-exchange resin for azo dye adsorption was 117 mg/g. Herein, authors have presented a plausible explanation for the results obtained in this experiment. Low adsorption capacity can be due to the high molecular structure of AB 113, which can make it difficult to penetrate the porous structure of the anion-exchange resin to arrive at functional groups [36]. 

For the adsorption of the persistent azo dye Acid Orange 10 (OG 10), a strongly basic anion-exchange resin IRA 400 (IRA 400) was employed. Herein, the adsorption capacity was 317.6 mg/g (25 °C and 65 min). Also, from the isotherm study, for the adsorption process of OG 10 on IRA 400, that adsorption capacity was found to be favorable [35]. 

### 4.2. Application of Ion-Exchange Resin in Biological Study

It is known that wastewater is treated by numerous and comprehensive methods, such as membrane filtration, sedimentation, precipitation, adsorption, electrocoagulation, electrochemical processes, ion exchange, oxidation, coagulation, flocculation, reverse osmosis, solvent extraction, electrolysis, reduction, and precipitation [6,10,93]. 

Although so many methods are available nowadays, all of them have advantages and disadvantages and suffer from different limitations, such as high energy consumption, serious pollution, and high cost [6,93].

Ion-exchange resins are alternatives or additions to the currently used methods for water treatment. Biological ion exchange (BIEX) has been proposed as an alternative to biological activated carbon (BAC) for removing natural organic matter, which comes from decomposed plant and animal matter and is a heterogeneous mixture of organic compounds [94,95]. Organic matter promotes bacterial growth in drinking water distribution systems and influences the color, odor, and taste of water [95]. Lee et al. used the BIEX+BAC system for the removal of organic carbon and trihalomethane formation potential and demonstrated that it is has an efficacy of almost 60% for removing dissolved organic carbon (59.9%) and assimilable organic carbon (61.2%) [95].

BIEX systems with different resins (magnetic ion-exchange resin (MIEX), non-magnetic resin (NIEX), polystyrenic resin (DIEX)), and one BAC system were tested on wastewater in order to remove dissolved organic carbon (DOC), and the anti-inflammatory drug ibuprofen (IBU) was selected as a model pollutant by Xu et al. Generally, DOC removal efficiency in BAC is around 20–30%, but higher DOC removal (80–96%) was achieved with synthetic wastewater containing glucose or polysaccharide as a carbon source [96,97,98,99]. In Xu’s study, DOC removal by the four systems was about 84%, while ibuprofen removal was nearly 100% (bio-MIEX), 60% (bio-NIEX), 61% (bio-DIEX), and 89% (BAC) [99]. 

One serious threat to the aquatic ecosystem is the presence of cyanobacterial toxins that are released as the cell dies, and they can persist in the water for up to three weeks after the algal bloom disappearance [100,101,102]. Cyanotoxin removal is limited due to the presence of NOM. The anion-exchange (IX) process, used as a cost-effective treatment for the removal of DOC, is a viable alternative for simultaneous NOM and cyanotoxin removal in bloom-laden waters, but it also has the potential to remove inorganic ions, such as sulphate and nitrate, which have been previously reported as strong competitors for NOM [102].

Nitrogen (N) and phosphorus (P) removal from wastewater is important because their presence can cause surface water eutrophication. Ion-exchange resins have been used for N and P removal. Using a macroporous ion-exchange resin, Zeng et al. showed that the resin had strong selectivity for NO_3_^−^. The resin denitrification pilot plant achieved stable and continuous operation for two months, with removal rates of 41.65%, 42.96%, 55.37%, 91.8%, and 90.81% for COD, TP, NH_4_^+^, -N, NO_3_^−^, -N, and TN, respectively. After the resin was regenerated, the removal rates of NO_3_^−^, -N, TN, and the regeneration recovery rate were above 90% [93]. Phosphate ion removal from aqueous solutions using a strong anion-exchange resin, Purolite A200E, is influenced by pH (highest phosphate removal occurred in the pH interval 7–9); it increases with the increase in resin dosage, stirring speed, and temperature and reduces with the increase in initial phosphate concentration and the presence of competing ions [103]. 

In Xiangdong Xu et al.’s study, the results of 30 exchange-regeneration cycles showed that zeolite (clinoptilolite), resin (strong acid cation resin), and MS (4A molecular sieve) can be used to separate and up-concentrate NH_4_^+^ from domestic wastewater; the resin exhibited the highest NH_4_^+^ capture ability and up-concentration properties with low resistance to Ca(II) and Mg(II) contamination [104]. 

Ion-exchange-resin-based fluoride removal processes are highly effective and capable of reducing fluoride concentration in water to below regulatory limits (as low as 0.7–1.5 mg/L); their effectiveness is dependent on a multitude of factors, including the resin type, fluoride concentration, water chemistry, presence of other ions, flow rate, contact time, and resin regeneration frequency [105]. 

The concern regarding the presence of heavy metals within the environment lies in the fact that these metallic elements are not biodegradable; instead, they tend to bioaccumulate, and they possess the ability to travel through the eco–system and reach human beings through the food chain. The ion-exchange method for the removal of heavy metals offers numerous advantages over other methods. The primary benefits of ion exchange are metal value recovery, selectivity, reduced sludge volume, and compliance with stringent discharge criteria [10,106]. 

In Mandal’s study, the tamarind triazine amino propanoic acid’s (TTAPA) resin removal efficiency was highest for Fe(II), followed by Cu(II), Zn(II), Pb(II), and Cd(II). The chelating ion-exchange resin also had a metal ion recovery of more than 95%. TTAPA acts as a flocculant and metal ion exchanger that helps in the sequestration of toxic and hazardous metal ions from industrial effluent [107]. 

Poly-γ-glutamic acid (PGA)-based composites are used to remove heavy metals from water (PGA–iron oxide for the removal of Cd(II), Ni(II), Cr(III), Cu(II), and Pb(II); PGA–graphene oxide for the removal of Cd(II), Ni(II), and Cu(II); PGA–natural biopolymers for the removal of Cd(II), Ni(II), Cu(II), Pb(II), and Co(II); PGA–inorganic material for the removal of Cu(II), Cr(III), and Pb(II); PGA–nanoparticles for the removal of Cd(II), Cu(II), and Ni(II) [108]. 

The Amberlite IRA400 anionic exchange resin is the best way to eliminate chromium metal from drainage water [106].

Cation-exchange resins with carboxyl (–COO^−^) or sulfonic (–SO_3_^−^) groups are effective for Ni(II) adsorption from Ni(II)-containing wastewater [109].

Chelating ion-exchange resins possessing bis-picolylamine functional groups are well suited for applications where the selective recovery of Cu(II) from highly acidic media (pH < 2) is required because these resins have good acid resistance due to their picolylamine-based structure. Lewatit MDS TP 220 chelating resin for Cu(II) has been studied, and it exhibited excellent Cu(II) adsorption and high selectivity in multimetal solutions with a pH of 1.5, owing to its strong affinity for Cu(II) [110].

Purolite^®^ C100, a strong acid cation-exchange resin, was used in experimental studies. The ion-exchange recovery of Cu approached 94.4%, while Pb recovery was 92.9% with a Purolite^®^ C100 resin dose range of 40 g to 80 g in the pH range of 3 to 12 [10]. Abo-Farha et al., using Purolite^®^ C100, obtained the maximum cation-exchange resin adsorption percent of 86.4%, 80.4%, and 75.5% for Ce(IV), Fe(III), and Pb(II) [111].

The applicability of resin-supported nano zero-valent copper (nZVC@D201) for the simultaneous removal of ciprofloxacin (CIP) and Cr(VI) was systematically investigated and is a promising alternative composite for the simultaneous removal of Cr(VI) and the degradation of CIP from wastewater (CIP and Cr(VI) removal rates of the nZVC@D201 composite were 78.7% and 95%, respectively) [112]. 

Norfloxacin’s (NX) different concentrations influence on a biological magnetic anion-exchange (B-MAEX) system activity on removing DOC and triclosan was analyzed, and it was established that the B-MAEX system is resistant to NX inhibition due to its higher biological activity [113].

## 5. Biological Methods Used for Biodegradation of Pollutants in the Environment

Population growth and industrialization triggered a constant increase in the amount and diversity of the chemical compounds. In spite of many domestic and industrial applications involving chemical compounds such as azo dyes, these chemical compounds end up in the domestic and industrial wastewaters and, subsequently, in the environment. 

The increasing presence and structural diversification of toxic compounds, as well as their longer residence time in the environment, is an issue not only in environmental science but also in public health [114]. Toxic substances from the azo dye class, released into the environment, can induce drastic changes at the biotope level. Many biological processes have been disturbed, from microorganisms to humans. Unfortunately, there are little data on the ecological risk to living organisms exposed to the presence of these toxic compounds, but increasing efforts are being made to determine the acute toxic effect of organic substances in water. 

Toxicity bioassays use biological systems (various biological models from bacteria to vertebrates) for the detection of toxic compounds in environmental samples and the assessment of their eco toxicity (e.g., wastewater effluents). The use of micro bioassays with a short exposure time ensures an assessment of acute lethal toxicity; therefore, micro bioassays are used as surveillance and warning tests [115]. Recently, more and more studies are being directed towards the toxic effect of active compounds on organisms in general as well as on the human organism in particular [116]. Given the complexity of the pollutants that are discharged into the environment today, removals based on strictly chemical criteria are no longer sufficient and appropriate; therefore, the use of beneficial biological organisms becomes inevitable in measuring and assessing the potential impact of contaminants on the environment. The legislative framework, such as Registration Evaluation and Authorization of Chemicals, should supervise the chemical production and commercialization in order to prevent a harmful effect on environmental and human health.

The potential risk assessment of these compounds could be tested by a wide range of methods [117] based on the chemical compound applicability (Table 4, below) [118]. 

The test results provide reliable acute and chronic toxicity data regarding their chemical risk [129].

The testing strategy of micro-biotests uses bacteria as test microorganisms for the toxicity assessment. The use of bacteria in ecotoxicity studies has several significant benefits, including (i) its associated metabolic complexity with a high capacity to adapt and interact with the environment and the rapid exchange of substances between the organism and the environment, which favors the survival of these microorganisms; (ii) its metabolic and physiological activities at the bacterial level being susceptible to toxic substances much faster than those in higher organisms; (iii) its reduced testing time due to the short growth/multiplication time of the bacteria.

Besides the ecotoxicological effect of the chemical compounds, their biodegradation plays an important role in maintaining environmental and human health. Domestic and industrial wastewater treatment plants (WWTPs) have become essential in environmental health. The WWTP biological treatment step is important and relies on bacterial communities. The activated sludge (formed by bacterial communities) is the main element in biodegrading pollutants. In spite of a toxic effect of pollutants, bacteria have a powerful adaptation mechanism, which overcomes most pollutants’ toxic effect; therefore, bacteria biodegrade most pollutants [120,121,122,123,124,125,126,127,128,129,130,131,132,133].

The biodegradation of azo dyes can be achieved under conventionally anaerobic conditions, facultatively anaerobic and aerobic, by different types of bacteria [134]. The bacterial mechanism used in the degradation of azo dyes involves the azo-reductive cleavage of the -N=N- bond under the influence of azo reductase enzymes, under anaerobic conditions, leading to colorless solutions and the production of potentially hazardous aromatic amines as a result of the breaking of these bonds. The resulting intermediates are further degraded aerobically or anaerobically [135,136]. Many scientific studies have focused on the use of bacteria for the removal of dyes from textile effluents [137,138,139,140]. The discharge of textile effluents into surface waters (rivers, lakes, and sewage networks) has reduced dissolved oxygen concentration, thus creating anoxic conditions that are lethal to resident organisms. In general, the microbial degradation of azo dyes involves electron transfer to the aromatic nucleus of benzene or naphthalene-type dye molecules, which behave as a final electron acceptor, leading to the formation of colorless aromatic amines, which are carcinogenic. Bacteria use the resulting amines as a carbon and energy source for their growth. Azo dyes respond to biological degradation when both aerobic and anaerobic conditions are used [141].

## 6. Conclusions

This review aims to present the fundamental principles, classifications, and current trends in the design and development of ion exchange in aqueous systems. Reducing environmental pollution by using different materials with ion-exchange properties is one of the current research trends because it involves the use of environmentally friendly technologies. So, ion-exchange resins are water insoluble, cross-linked, and chemically inert materials, obtained from the copolymerization of styrene with divinylbenzene and on the structure of which functional groups are grafted that lead to an increase in adsorption capacity.

Also, ion-exchange systems represent accessible materials for wastewater depollution. 

The use of ion exchangers continues to be the most convenient method for the purification of polluted wastewater when large volumes of effluent containing trace metal ions need to be treated over conventional methods, which are not suitable.

Ion-exchange equilibrium allows almost all ionic species to be removed from a solution, regardless of how highly concentrated they are found to be. 

Thus, all compounds that contain ionizable groups in their structure can be retained by an ion-exchange mechanism from the solution, regardless of their concentration.

For pollutants from the class of azo dyes, ionic groups ensure their solubility and the possibility of retention through an ion-exchange mechanism on materials with ion-exchange properties.

The procedure for retaining compounds with high molecular mass on ion-exchange resins through ion-exchange balance is applied at the industrial level. Studies regarding the use of conventional anion-exchange resins, as well as a polymer resin modified with analytical reagents, allow selective separation as well as the preconcentration of metal cations at the trace level in the solution. These resins with complex properties are obtained by immobilizing some of the reaction’s ion exchange or physical retention by this promising mechanism compared to the complex-forming resins established by direct synthesis.

## Figures and Tables

**Table 1:** Optimum pH range for cation and anion-exchange resins required in analytical applications (adapted according to reference [62]).

Type of Resins	Type of Functional Grouping	pH Range
Cation-exchange resins	Weakly acidic	9–14
	Strongly acidic	0–14
Anion-exchange resins	Weakly basic	0–9
	Strongly basic	0–14

**Table 2:** Relationship between the percentage of divinylbenzene and water content (adapted according to reference [68,69]).

Type of Resin	DVB (%)	Water Percentage (%)
Microporous (gel)	2	80
	4	70
	8	55
Macroporous (macroreticular)	12	45
	16	40
	20	50–65

**Table 3:** Theoretical and operational exchange capacity of ion-exchange resins (adapted according to reference [68]).

Type of Resin		Total Exchange Capacity eq/L	Operating Capacity eq/L
Weakly acid	Anion-exchange resin	3.7 ÷ 4.5	1.0 ÷ 3.5
Strong acid		1.7 ÷ 2.2	0.6 ÷ 1.7
Weakly basic	Anion-exchange resin	1.1 ÷ 1.7	0.8 ÷ 1.3
Strongly basic		0.9 ÷1.4	0.4 ÷ 0.9

**Table 4:** Biological tests used in INCD ECOIND, Bioassay-Biological Analysis Laboratory (adapted according to reference [118]).

Species	Test	Matrices	Type of Test	Endpoint Effect	Test Period /Incubation
Freshwater fish					
*Cyprinus carpio*/ *Carassius auratus gibelio*	OECD 203 * SR EN ISO 7346-1 [119]	Chemical/water samples	Acute	Mortality, LC50, clinical signs	96 h, 21–22 °C
	In-house procedure	Chemical/water samples	Chronic	Growth instant rate, mortality rate, biomass mean, production, used food rate, and biochemical indicators—hepatic enzyme activity, MATC	30–90 days, 21–22 °C
	In-house procedure	Chemical/water samples	Acute or chronic	Enzymatic activity of antioxidant enzyme, histopatological modification	>96 h, 21–22 °C
	OECD 305 ** (Laboratory tests)	Metals	Acute or chronic	Bioconcentration factor (BCF)	>96 h, 21–22 °C
Aquatic plants					
*Spirodela polyrhiza*	ISO 20227 Spirodela Duckweed Toxkit [120]	Chemical/water samples	Acute	Growth inhibition, EC50	72 h, 25 °C
Planktonic Crustacean					
*Daphnia magna*	OECD 202 ***, Daphtoxkit F	Chemical/water samples	Acute	Mortality/immobilization LC50	24–48 h, 20 °C
Benthic Crustacean					
*Heterocypris incongruens*	ISO 14371 Ostracodtoxkit F [121],	Chemical/water samples/sediments	Chronic	Mortality/growth inhibition, LC50	6 days, 25 °C
Green Algae					
*Selenastrum capricornutum/* *Pseudokirchneriella subcapita*	OECD 201 ****, Algaltoxkit F	Chemical/water samples	Acute/chronic	Growth rate inhibition/biomass inhibition, EC50	72 h, 21–25 °C
Rotifers					
*Brachiounus calyciflorus*	ASTM Standard Guide E1440-91, Rotoxkıt F [122]	Chemical/water samples	Acute	Mortality, EC50	24 h, 25 °C
Protozoan					
*Tetrahymena thermophila*	Protoxkıt F	Chemical/water samples	Chronic	Reproduction inhibition, EC50	24 h, 30 °C
Bacteria					
Marine luminescent bacteria—*Aliivibrio fischeri*	DIN EN ISO 11348-3 BioFix Lumi [123]	Chemical/water samples	Acute	Luminescence inhibition, IC50	15/30 min, 20 °C
*Microbacterium* sp., *Brevundimonas diminuta*, *Citrobacter freundii*, *Comamonas testosterroni*, *Enterococcus casseliflavus*, *Delftia acidovorans*, *Kurthia gibsonii*, *Sthaphilococcus warnerii*, *Pseudomonas aurantiaca*, *Serratia rubidae*, *Pichia anomalia*	MARA test (Microbial Array for toxicity Risk Assessment)	Chemical/water samples	Acute	Microbial growth inhibition, MTC	18 h, 30 °C
*Escherichia coli*	Geno toxicity test SOS Chromotest	Chemical/water samples	Acute	Effects on gene expression (β-galactosidase)	18 h, 37 °C
Terrestrial plants					
*Lepidium sativum*, *Sinapis alba*, *Sorghum saccharatum*	SR EN ISO 11269, Phytotoxkit [124]	Chemical, soil, sludge, waste, sediment, water	Acute	Germination and root growth inhibition, EC50	72 h, 25 °C

Where: OECD is Organization for economic cooperation and development; * OECD Guidelines for the Testing of Chemicals, Section 2 Test No. 203: Fish, Acute Toxicity Test (OECD 203) [125]; ** OECD 305: Bioaccumulation in Fish: Aqueous and Dietary Exposure [126]; *** ECD Guidelines for the Testing of Chemicals, Section 2: Effects on Biotic Systems Test No. 202: Daphnia sp. Acute Immobilisation Test [127]; **** OECD Guidelines for the Testing of Chemicals, Section 2, Effects on Biotic Systems, Test No. 201: Freshwater Alga and Cyanobacteria, Growth Inhibition Test [128].
